# Conserved Secondary Structures in Aspergillus

**DOI:** 10.1371/journal.pone.0002812

**Published:** 2008-07-30

**Authors:** Abigail Manson McGuire, James E. Galagan

**Affiliations:** The Broad Institute of M.I.T. and Harvard, Cambridge, Massachusetts, United States of America; University of Western Cape, South Africa

## Abstract

**Background:**

Recent evidence suggests that the number and variety of functional RNAs (ncRNAs as well as cis-acting RNA elements within mRNAs ) is much higher than previously thought; thus, the ability to computationally predict and analyze RNAs has taken on new importance. We have computationally studied the secondary structures in an alignment of six *Aspergillus* genomes. Little is known about the RNAs present in this set of fungi, and this diverse set of genomes has an optimal level of sequence conservation for observing the correlated evolution of base-pairs seen in RNAs.

**Methodology/Principal Findings:**

We report the results of a whole-genome search for evolutionarily conserved secondary structures, as well as the results of clustering these predicted secondary structures by structural similarity. We find a total of 7450 predicted secondary structures, including a new predicted ∼60 bp long hairpin motif found primarily inside introns. We find no evidence for microRNAs. Different types of genomic regions are over-represented in different classes of predicted secondary structures. Exons contain the longest motifs (primarily long, branched hairpins), 5′ UTRs primarily contain groupings of short hairpins located near the start codon, and 3′ UTRs contain very little secondary structure compared to other regions. There is a large concentration of short hairpins just inside the boundaries of exons. The density of predicted intronic RNAs increases with the length of introns, and the density of predicted secondary structures within mRNA coding regions increases with the number of introns in a gene.

**Conclusions/Sigificance:**

There are many conserved, high-confidence RNAs of unknown function in these *Aspergillus* genomes, as well as interesting spatial distributions of predicted secondary structures. This study increases our knowledge of secondary structure in these *aspergillus* organisms.

## Introduction

Recent experimental evidence in mammals has indicated that the portion of the genome that is transcribed, as well as the number of functional RNAs in the genome, is much higher than previously thought [1]–[10]. Functional RNAs include noncoding RNAs as well as cis-acting RNA elements within mRNAs. The number of known roles for RNA is increasing rapidly, including gene regulation by microRNAs [11], [12], regulation by cis-acting translational control elements within mRNAs such as riboswitches [13], X-chromosome inactivation by Xist [14], as well as many others [15]. Therefore, predicting the locations of functional RNA elements (both ncRNAs as well as cis-acting RNA elements) has taken on new importance. Despite the wide availability of fungal genome sequences, no thorough computational analysis of RNA secondary structures has been conducted in fungi.

Several methods have been used for identifying functional RNAs in genome sequences, taking advantage of information contained in genome alignments [8], [16]–[23]. EvoFold [8] and QRNA [18] use Stochastic Context Free Grammars (SCFG's). RNAz [19] is based on RNAalifold [24] and the Vienna RNA package [25], [26], which evaluate the folding thermodynamics. Several whole-genome computational searches for functional RNAs have been performed: using EvoFold on human [8], [27]; RNAz on human [9], [27], nematodes [28], and *Ciona* [29]; and QRNA on *S. cerevisiae* [30] and *E. coli* [18]. Will et al. have clustered the resulting predictions in *Ciona* by structural similarity [31].

Here we report the results of using RNAz to perform a genome-wide analysis of secondary structures in six aspergillus genomes. RNAz is a fast algorithm which has been used successfully in several whole-genome searches for predicted secondary structures [9], [27]–[29]. The RNAz algorithm uses the RNAfold and RNAalifold [24] programs to find the minimum free energy structure for each individual sequence in the alignment, as well as the minimum free energy of the consensus structure for the alignment, including a “covariance term” which takes into account compensatory and consistent mutations which preserve the RNA secondary structure. Compensatory mutations involve changing both members of a base pair (i.e. GC→AT) to preserve the secondary structure, whereas consistent mutations involve a change to only one member of the pair while preserving secondary structure (i.e. GC→GU). RNAz compares the minimum free energy of the predicted structure to that of random sequences of the same base composition to calculate a z-score, which is an index of the thermodynamic stability of the structure. It also calculates a “structure conservation index” (SCI), which is the ratio of the free energy of the consensus structure to the average of the free energies of the individual sequences. A high value for this corresponds to a conserved structure. The z-score, SCI, and % sequence identity are used as input to an SVM (trained on known RNA alignments from Rfam) which decides the likelihood of the alignment being a functional RNA.

Previous whole-genome searches using RNAz [9], [27]–[29] have found large numbers of predicted secondary structures with false positive rates estimated between 16 and 70%. A study in the human genome[9] predicted 30,000 secondary structures, including 10,000 conserved across vertebrates. A number of new microRNAs were predicted, as well as a large number of predicted secondary structures that did not fit into groups of known RNA structures.

We used RNAz to search for sequences likely to form conserved secondary structures in an alignment of six *Aspergillus* genomes, and then cluster the predicted secondary structures by structural similarity to find structural classes. Our alignment covers approximately 60% of the *A. nidulans* genome; hence we are only scanning 60% of the genome in our study.

We find over 7000 predicted secondary structures, including a new ∼60 bp hairpin motif found primarily inside introns. Different genomic regions primarily contain different structural classes of predicted secondary structures. Exons contain primarily long, branched hairpins, 5′ UTRs primarily contain groupings of short hairpins located near the start codon, and 3′ UTRs contain very little secondary structure compared to other regions. There is a large concentration of short hairpins just inside the boundaries of exons (gene starts, gene stops and splice sites). In addition, the density of predicted intronic RNAs increases with the length of introns, and the density of predicted secondary structures within mRNA coding regions increases with the number of introns in a gene.

## Methods

### Whole-genome alignments

We analyzed *Aspergillus nidulans*, *Aspergillus oryzae*, *Aspergillus fumigatus*, *Aspergillus terreus*, *Aspergillus clavatus*, and *Neosartorya fischeri*. *Aspergillus flavus* was included when the alignments were constructed, but discarded for the RNAz searches because RNAz takes a maximum of six sequences in its input alignment. *Aspergillus flavus* was discarded because it had the poorest assembly and is very similar to *A. oryzae*. Complete genomes were available for *A. nidulans* [32], *A. oryzae* [33], and *A. fumigatus* [34]. For the other four genomes, incomplete genome assemblies were used.

*A. nidulans* was used as a reference in constructing the multiple alignment. Pairwise whole genome alignments were done using Patternhunter [35]. Colinear blocks were then identified and aligned with Lagan [36]; multiple alignments [37] were constructed with Mlagan [36]. 40% of the *A. nidulans* genome was covered by multiple alignment of all 7 genomes. 60% of the *A. nidulans* genome was covered by multiple alignment of 2 more genomes. Sequences within the alignments consisting largely of gaps were filtered out, as in Washietl et al., 2005 [9].

### Searches for secondary structure using RNAz

We searched these alignments for regions likely to form conserved RNA secondary structures using RNAz [9], [19]. We used 400 bp search windows (tiled every 100 bp across the whole genome alignment, for a total of 250,268 successful search windows) as well as 200 bp search windows (tiled every 40 bp for a total of 410,998 successful search windows). We chose a longer search window size than the 120 bp windows used previously by Washietl et al. [9] because 120 bp windows were not adequate to identify several of the few known RNAs in aspergillus. We found that 200 bp windows are not too long to correctly find shorter structures as well. An RNAz cutoff of 0.5 was used to select search windows with predicted secondary structure for further analysis. Unlike the previous search using RNAz by Washietl et al. [9], exonic sequence was kept within our search space.

To determine which of the predicted secondary structures correspond to known RNAs, we searched these sequences against the Rfam database [38]. A loose BLAST [39] search was used to determine possible candidate matches, followed by a more careful search on possible hits using Infernal [40]. tRNA-ScanSE [41] was used to search for tRNAs.

To calculate false positives, we used the script shuffle-aln.pl [22] to shuffle each search window; then we searched the shuffled sequences with RNAz. This conservative shuffling procedure generates random alignments, preserving length, base composition, overall conservation, local conservation, and gap pattern.

### Structural classes

To calculate structural similarity between hits, we used RNAdistance [26], which calculates a tree or string edit distance between RNA structures. At the time when we performed this analysis, we found RNAdistance to be the most useful and practical tool available for this purpose, despite issues relating to the treatment of sequences of dissimilar length and the fact that RNAdistance performs a global, rather than local, alignment. Since our analysis was performed, an improved local alignment tool called LocARNA has been published and applied to whole-genome RNAz searches in *Ciona* [31].

We calculated all-vs.-all RNAdistance values, using all four of the RNAdistance structure representations (full, HIT, weighted coarse, and coarse). A simple hierarchical clustering algorithm was used to cluster these motifs by their RNAdistance values. This clustering was performed separately for each of the RNAdistance structure representations, resulting in four sets of structural classes. Fixed cutoffs were used in the clustering based on RNAdistance values. For each cluster, we then calculated p-values for overrepresentation of functional groups and regions of the genome (using the hypergeometric function). We calculated p-values for overrepresentation in COG functional group categories, introns, exons, 5′ UTRs, 3′ UTRs, and noncoding regions, as well as overlaps between 5′UTRs and exons, 3′ UTRs and exons, and introns and exons.

We then checked to see if the known RNAs found by RNAz were clustering together. The tRNAs were the largest group of knowns found by RNAz, and these grouped nicely into several clusters. When looking at the other known RNAs with >1 instance found by RNAz (5S rRNA, TPP riboswitch, U6 spliceosomal component), we saw that different structures are associated with quite different RNAdistance values; hence no single RNAdistance cutoff was adequate for defining the clusters. Therefore, the second way we created clusters was to calculate p-values when each new member was added and to select those clusters with minimal p-values. We sorted the clusters by p-value and applied cutoffs: p<1e-7 (includes correction for multiple hypothesis testing), and N<500 (number in cluster).

### Predicting intronic branch sites

To predict the locations of branch sites in introns, the regions from 10–30 bp upstream of the 3′ splice site were aligned in all annotated introns using AlignACE [37]. Since this only identified a motif in one quarter of the introns, we used the loose consensus pattern RYURAY (seen in the motifs found by AlignACE) and picked the 3′-most instance in each intron.

### Searching for miRNAs

To search for possible animal-like miRNAs, we selected conserved hairpins and examined them using MiRscan [44]. To search for possible plant-like miRNAs, we selected conserved hairpins, and then looked to see which had possible conserved miRNA targets, allowing up to 4 mismatches within exons. To search for targets, we used Patscan [45] to do searches over the sequences of COGs plus 1000 bp upstream and downstream for each possible miRNA. Hits should be in exons for plant-like miRNAs.

## Results and Discussion

We generated a multiple alignment of diverse *Aspergillus* genomes with an average pairwise sequence identity of 58%, which is close to the optimal level of sequence identity for searching for RNAs. If the genomes were more similar, there would not be sufficient consistent and compensatory mutations observed to infer the presence of base-pairing; if the genomes were less similar, there would not be a good enough alignment to infer structure. Washietl and Hofacker [22] plotted the average z-scores of structural and sequence-based pairwise alignments of SRP RNAs versus pairwise identity and showed that there is a peak in the z-scores for sequence-based alignments around 60% average pairwise sequence identity; z-scores dropped off for both higher and lower levels of sequence identity.

**Figure 1:**
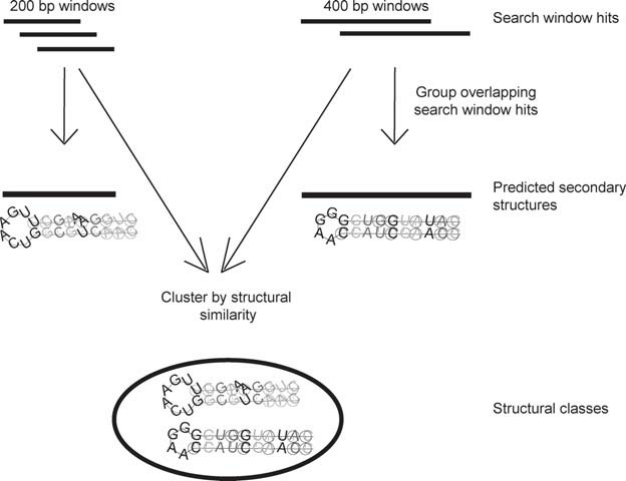
Obtaining predicted secondary structures and structural classes. Overlapping search window hits are grouped into predicted secondary structures. Since most predicted secondary structures are primarily contained within a single search window, we clustered search window hits by structural similarity into structural classes.

We searched our whole-genome alignments with RNAz using 200 bp and 400 bp long search windows. Using a 200 bp long search window, 2.4% of the search windows (9663 windows) yielded hits with RNAz score >0.5; using the 400 bp search window, 4.0% of the search windows (9916 windows) resulted in hits with RNAz score >0.5 (see Table 1). These search window hits were grouped into non-overlapping predicted secondary structures (see Figure 1). Using the less stringent RNAz cutoff of 0.5, and only requiring conservation in two more organisms, results in 5517 predicted secondary structures using the 200 bp search window, and 5479 predicted secondary structures using the 400 bp search window (see Table 2). Using the more stringent RNAz cutoff of 0.9, and requiring conservation in all six organisms, yields 326 high-confidence predicted secondary structures using the 200 bp searches and 398 using the 400 bp search window. There is a great deal of overlap between the results found using the 200 bp and 400 bp search windows. Combining all of the hits, from both the 200 bp and 400 bp windows, for the less stringent RNAz cutoff together gives us 19579 search window hits with RNAz score >0.5 in 7450 non-overlapping predicted secondary structures. We used this combined group of 19579 hits for further analysis, including clustering by structural similarity (see Figure 1).

**Table 1:** Summary of RNAz searches by region of genome.

	# windows searched	RNAz score>0.5		RNAz score>0.9	
		# hits	fraction of windows w/hits	# hits	fraction of windows w/ hits
a. 200 bp windows					
Intron	8917	346	1.9e-2	90	5.1e-3
Overlaps splice site	78893	1596	1.0e-2	352	2.2e-3
Noncoding	47826	1786	1.9e-2	384	4.0e-3
Exon	182381	2065	5.7e-3	415	1.1e-3
5′ UTR	37206	1625	2.2e-2	402	5.4e-3
3′ UTR	24094	766	1.6e-2	184	3.8e-3
Overlaps start	17937	1051	3.0e-2	293	8.2e-3
Overlaps stop	13744	428	1.6e-2	100	3.6e-3
Totals	410998	9663	2.4e-2	2220	5.4e-3
b. 400 bp windows					
Intron	4884	214	2.2e-2	22	2.3e-3
Overlaps splice site	50542	1902	1.9e-2	199	2.0e-3
Noncoding	43639	1344	1.5e-2	160	1.8e-3
Exon	63415	1093	8.6e-3	139	1.1e-3
5′ UTR	29873	1322	2.2e-2	122	2.0e-3
3′ UTR	20075	560	1.4e-2	82	2.0e-3
Overlaps start	20241	2301	5.7e-2	276	6.8e-3
Overlaps stop	17599	1180	3.4e-2	88	2.5e-3
Totals	250268	9916	4.0e-2	1088	4.3e-3

**Table 2:** Clustering of RNAz hits into predicted secondary structures.

Conserved in all 6 genomes	200 bp windows		400 bp windows	
	RNAz score>0.5	RNAz score>0.9	RNAz score>0.5	RNAz score>0.9
# predicted secondary structures^a^	1259	326	3651	398
# groups in shuffled controls^b^	384	57	1423	76
False positives^c^	31%	17%	39%	19%
Length of predicted secondary structures	191,960	50,072	657,680	94,832
Length of hits in shuffled controls	52,889	8,048	276,294	15,018
Fraction of *A. nidulans* genome	0.64%	0.17%	2.2%	0.32%

Conserved in 2 or more genomes	200 bp windows		400 bp windows	
	RNAz score>0.5	RNAz score>0.9	RNAz score>0.5	RNAz score>0.9
# predicted secondary structures^a^	5517	1313	5479	624
# groups in shuffled controls^b^	1916	277	2170	128
False positives^c^	35%	21%	40%	21%
Length of predicted secondary structures	835,065	209,146	1,191,771	174,609
Length of hits in shuffled controls	271,100	40,321	484,903	29,797
Fraction of *A. nidulans* genome	2.8%	0.69%	4.0%	0.57%

a number of non-overlappings groupings of RNAz hits on native sequence.

b number of non-overlappings groupings of RNAz hits found on an equivalent amount of shuffled control sequence.

c false positives based on number of predicted secondary structures: number of groups in shuffled controls divided by the number of predicted secondary structures.

### Calculating false positive rates using searches over shuffled sequence

Since a complete reference set of secondary structures in *Aspergillus* is not available, we must estimate the rate of false positives by comparing the observed number of predicted secondary structures with the number that we would expect to occur by chance. Our false positive rate is based on the number of final, non-overlapping predicted secondary structures (see Figure 1). The process of grouping overlapping search window hits (shown in Figure 1) was repeated on the shuffled search window hits to obtain a set of “shuffled predicted secondary structures”. The false positive rate is computed by dividing the number of predicted secondary structures by the number of “shuffled predicted secondary structures” obtained on shuffled sequence. There are fewer false positives for our more stringent (RNAz score >0.9) threshold. As expected, the rate of false positives is higher for searches performed using the 400 bp search windows (39% for RNAz score >0.5 and 19% for RNAz score >0.9) than for searches performed using the 200 bp search windows (31% for RNAz score >0.5 and 17% for RNAz score >0.9). Table 2 also shows that the predicted secondary structures found in native sequence are longer than those on shuffled sequence.

High false positives have also been reported in previous whole-genome searches for predicted secondary structure [8], [9], [27], [29]. In their search over the human genome using RNAz, Washietl et al. report false positive rates of 28.9% (RNAz score >0.5) and 19.2% (RNAz score >0.9) [9], which are similar to the values that we obtained for our 200-bp search windows, despite our search windows being longer in order to be able to identify known RNAs in *Aspergillus* (200 bp rather than 120 bp).

We believe that an adequate method of constructing proper controls is needed. Our shuffling method frequently does not remove the signal, since the shuffled sequence often still has an RNAz score above our cutoff threshold. This is in agreement with previous observations by Washietl et al. [9]. The number of possible permutations within this conservative shuffling procedure can be small, and the total amount of compensatory and consistent mutations will be preserved in the shuffled sequence. However, as discussed in Washietl et al. [22], a stronger shuffling algorithm disrupts the sequence enough to not be a meaningful control. 41% of our shuffled hits with RNAz score >0.5 overlap an unshuffled hit. So perhaps as many as 41% of the shuffled hits represent cases where the folding signal was simply not destroyed by shuffling.

In addition, a recent study by Babak et al. [42] showed that preserving dinucleotide frequencies, which we do not attempt to preserve in our shuffling strategy, is important and increases false positive rates in pairwise alignments. However, preserving dinucleotide frequencies in our multiple alignments can't be adequately performed while still preserving gap structure and patterns of conservation.

### Calculating sensitivity

Within our alignments, there are 78 known RNAs. Among our predicted secondary structures, we found matches to 63 of these (including 51 tRNAs, two TPP riboswitches, five 5S rRNAs, two U6, one U5, and one U2 spliceosomal RNA, and a U14 small nucleolar RNA), giving us an overall sensitivity of 81%. Since only approximately 200 RNAs have been identified in *A. nidulans*, [32], this represents a sizeable fraction of the RNAs already identified (see Table 3). Many of those that were not found are absent due to the fact that they were not aligned in our colinear blocks, which cover approximately 60% of the *A. nidulans* genome. Some other classes of RNAs evolve too quickly to identify significant conservation across the large evolutionary timescale in our dataset.

**Table 3:** Known functional RNAs found in *A. nidulans*.

	Previously Identified^1^	Identified by RNAz^2^	Contained in our alignments	Sensitivity^3^
tRNA	179	51	61	84%
5S rRNA	31	5	9	56%
U2	2	1	1	100%
U5	1	1	1	100%
U6	3	2	2	100%
U14	1	1	1	100%
Rnase P	1	-	-	-
SRP RNA	1	-	-	-
TPP riboswitch	3	2	3	66%

1 From Pain and Griffiths-Jones, 2005 (Galagan, Calvo, et al., 2005).

2 Identified tRNAs using tRNA-ScanSE, and other RNAs using Infernal with Rfam.

3 The number identified by RNAz divided by the number of *A. nidulans* knowns completely contained within the alignments input to RNAz.

### Preference for the coding strand

We calculated an association statistic [8] used to assess strand bias (see Table 4). In agreement with previous observations [8], we found a significant preference for motifs within mRNA-associated regions of the genome to be found on the coding strand (see Table 4). The difference between the coding and noncoding strands is primarily due to the presence of non-Watson-Crick “GU” base pairs in RNA (but not its reverse complement “CA”). We observed that the preference for the coding strand was most pronounced for motifs that overlap the start codon: for this region, there were 2.3 times as many hits on the forward strand for searches using 200 bp search windows and 2.4 times as many for searches using 400 bp search windows (see Supplementary Information). In contrast, when looking at hits in noncoding regions, there were slightly less hits on the forward strand (in relation to the closest gene) than the reverse strand (see Supplementary information). Unlike previous results in [8] in human, we saw no significant bias for coding strand 3′ UTRs motifs; the strongest bias was in 5′ UTRs and in folds overlapping the start codon.

**Table 4:** Strand bias.

	RNAz score>0.5			RNAz score>0.9		
	Avg. strand preference score^1^	#regions	p-value (association statistic) 1	Avg. strand preference score^1^	#regions	p-value (association statistic) 1
Intron	0.554	327	0.030	0.547	86	0.23
Overlaps a splice site	0.567	1005	1.2e-5	0.561	239	0.035
Exon	0.530	2631	1.2e-3	0.572	537	5.0e-4
5′ UTR	0.593	1352	3.8e-12	0.603	341	7.1e-5
3′ UTR	0.520	648	0.18	0.432	162	0.96
Overlaps start codon	0.685	504	3.8e-17	0.767	159	4.2e-12
Overlaps stop codon	0.484	190	0.69	0.511	45	0.5
Total	0.552	8054	5.1e-21	0.566	1862	6.5e-9

1 The strand preference score and association statistic was calculated in a manner similar to Pedersen et al. (2005)[8]. RNAz scores were evaluated on both strands. Each position was assigned a strand preference score depending on if the higher score was on the sense strand (strand preference score = 1), the antisense strand (strand preference score = 0), or if the scores on the two strands were equal (strand preference score = 0.5). This association statistic was assumed to be binomial distributed with parameter p = 0.5. The alternate hypothesis is that p deviates from 0.5.

### Clustering motifs by structural similarity

To identify groups of related predicted secondary structures, we clustered all of the search window hits by structural similarity (see Materials and Methods) to identify structure-based classes. Structural classes of predicted secondary structures are available at http://www.broad.mit.edu/ftp/pub/seq/msc/pub/aspergillus_folding/. Another study clustering the results of a whole-genome RNAz search in *Ciona* by structural similarity has recently been published [31]. Like this previous study, we were able to recover tRNAs as a structure-based class, in addition to identifying new classes of predicted secondary structures.

The entire clustering process (see Figure 1) was also repeated on shuffled controls. Because there were fewer hits in the shuffled controls than in native sequence, the shuffling process was repeated several times in order to generate several sets of “shuffled hits”, in order to have a number of shuffled hits equal to the number of native search window hits. We clustered both the native and the shuffled search window hits, and compared the resulting structure-based classes from native and shuffled sequence.

For each structure-based class, we calculated over-representation for each region of the genome. We applied p-value cutoffs based on this overrepresentation for regions of the genome (p<1e-7), and we required that the number of search window hits in a cluster be less than 500, to rule out nonspecific clusters. We found that native, unshuffled structure-based classes were much more over-represented for specific regions of the genome than shuffled structure-based classes. 97 unshuffled groupings make these cutoffs, whereas only 19 shuffled groupings make these cutoffs, and the p-values are much lower for the unshuffled ones (see Supplementary Information; Figure S1).

### Characteristic motifs by region of the genome

**Figure 2:**
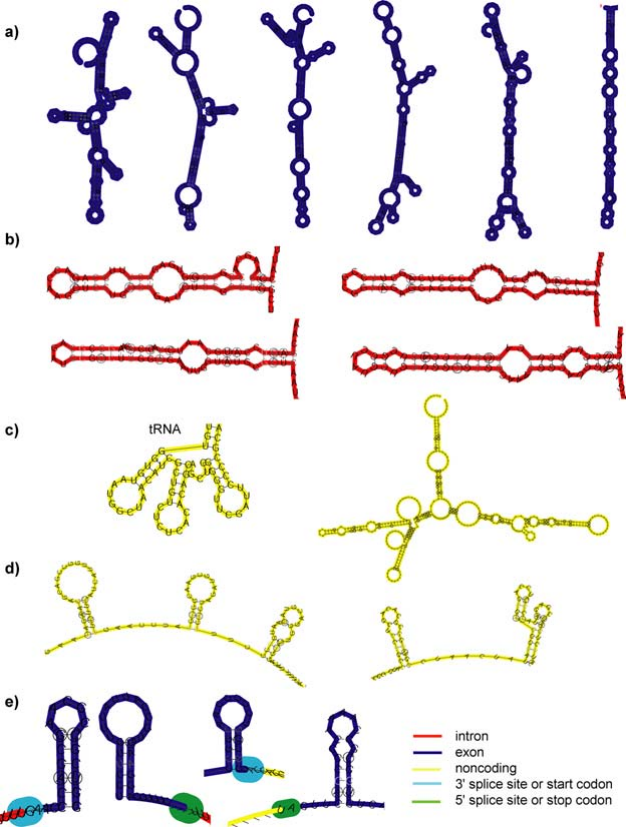
Examples of predicted secondary structure motifs by region of genome. a) Examples of long, branched hairpins found in exonic regions; b) New bulgy hairpin motif found in intronic regions; c) Examples of known or predicted noncoding RNAs found in intergenic regions; d) Examples of short hairpins found in 5′ UTR regions; e) Examples of short hairpins found just inside exons near exon boundaries (the most common type of motif in this region). Very few motifs were found in 3′ UTR regions.

In the structure-based classes described above, we found that different regions of the genome contained quite different motifs (see examples in Figure 2, as well as on the website). Clusters overrepresented in exons contain long structures, including many long hairpins. This is in agreement with searches across the human genome using EvoFold [8], which also yielded a surprising number of long folds that overlap coding regions. The presence of substantial amounts of secondary structure within exons agrees with the findings of Katz and Burge [47], who showed computationally that coding region sequences show a greater bias towards forming local RNA structures (as shown by folding free energy) than their shuffled counterparts. Other computational studies have predicted the presence of extensive secondary structure in coding regions [17], [48], [49]. Secondary structures overlapping coding regions are interesting because they are often involved in genetic recoding, and involve the dual constraints of codons and RNA structure. However, these long hairpins we identified do not contain more rare codons than expected. Examples of known uses of secondary structure within coding regions include signals for selenocysteine insertion [50], frameshifting [51], and RNA editing [52]. RNA structure can also modulate rates of translation in order to allow for proper protein folding.

Upstream 5′ UTRs preferentially contain short hairpins (often multiple short hairpins with intervening unstructured regions). Many of these groupings of short hairpins over-represented in 5′ UTRs exhibit positional bias. We calculated positional bias, using the binomial distribution, for each 50 and 100 bp window between 0 and 500 bp upstream of the start site. Several clusters exhibited bias for the region of the 5′ UTR closest to the start codon (0–50 bp or 0–100 bp upstream of the start codon). These clusters contained mostly two or three short hairpins separated by unpaired linkers.

In contrast to 5′ UTRs, there were no structural classes over-represented in 3′ UTRs. We also observed a lower density of high-scoring search windows in 3′ UTRs than in 5′ UTRs (see Table 1). This is surprising, because a previous search over the human genome using RNAz [9] found roughly equal amounts of predictions in 3′ and 5′ UTRs. Another previous search for functional RNAs over the human genome using EvoFold [8] found many more high-scoring motifs in 3′ UTR regions than in 5′ UTR regions. This previous study also showed that 3′ UTRs have greater bias for the coding strand than 5′ UTRs, which is also the opposite of what we observe (see Table 4 and Table S1). Our results could indicate that RNA structure in 3′ UTRs is not as important in fungi as it is in human genomic sequence. Consistent with this, human 3′ UTRs are also significantly longer than fungal 3′ UTRs [53]. It is also possible that RNAz is not able to detect 3′ UTR sequences as well as EvoFold, since EvoFold is more sensitive on AU-rich sequences, and RNAz is more sensitive on GC-rich sequences [27].

Several interesting motifs were also found entirely within noncoding regions, including several clusters of known tRNAs. There were also several other small clusters of long predicted secondary structures which are candidates for novel RNA genes (see Supplementary Information).

No convincing plant or animal microRNAs were found in *Aspergillus*, despite the fact that fungi branch from animals, and both animals and plants have microRNAs. Since no miRNAs have been previously identified in these fungi, it is not clear whether fungi have miRNAs; and if they do, whether their miRNAs would resemble animal or plant miRNAs. Fungi have RNAi [46], but to date no evidence has been reported indicating that this system has been adapted for use with microRNAs.

### Motifs found in introns

Among the groups over-represented in introns, there is an interesting motif: an approximately 60 bp long bulgy hairpin (see Figure 2b). The structural classes containing this motif (one cluster was obtained from each of the four clustering methods) are highly enriched for introns (p<1e-13). Intron lengths in aspergillus follow a very tight distribution, peaked at around 65 bp. However, the introns containing this motif average 183 bp in length. Therefore, a possible role for this motif is to decrease the effective length of the intron or to more efficiently bring together the splice sites and/or branch point for splicing efficiency. Another possibility is that this motif positions the branch site for interaction with the U2 snRNP. There are several known examples of hairpins affecting splicing efficiency [54], [55]. Another possible role for this motif is regulation of alternative splicing. This hairpin could serve as a protein binding site, or change the relative distances of the splice site and branch point, or of intronic or exonic splicing enhancers or repressors (ESEs/ISEs). There are several known examples of intronic hairpins that serve as probable or known binding sites for proteins involved in regulating alternative splicing [56], [57], or that regulate alternative splicing by other means [58], [59]. For example the istem in *Drosophila* is very similar in length and appearance to the motif we observe in aspergillus [56].

**Figure 3:**
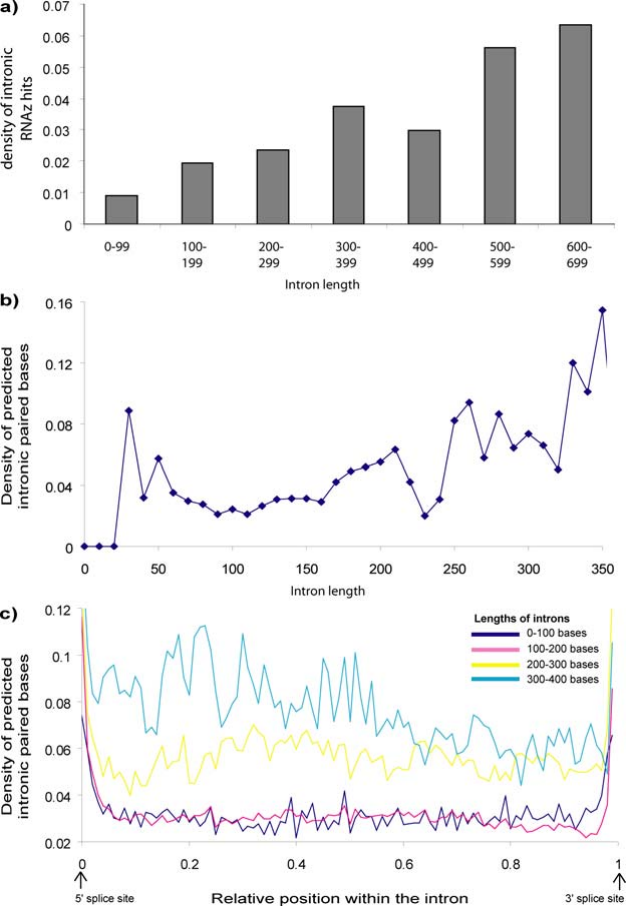
Longer introns have more predicted secondary structure. a) The density of hits (the number of RNAz hits with RNAz score >0.5 divided by the total number of windows searched) is plotted against the length of the intron. You can see that longer introns have a higher density of RNAz hits. b) The density of predicted paired bases also increases with the length of the intron. c) The density of predicted paired bases is plotted as a function of the relative position within the intron, for four different length groups of introns. You can see that longer introns (light blue and yellow curves) have a higher density of predicted paired bases across their entire length than shorter introns (the dark blue and pink curves).

There is also a great deal of other secondary structure within introns. In support of the idea that intronic secondary structure can serve to effectly shorten the distance between splice sites in introns that are longer than optimal, we observe that the density of predicted secondary structure increases with the length of the intron. This true for the density of intronic RNAz hits (see Figure 3a), as well as the density of predicted paired bases (see Figure 3b). Figure 3c shows the density of predicted intronic base pairs as a function of the relative position across the intron. It can be seen that longer introns have greater density of secondary structure across their entire length than shorter introns. The same relationships hold true when looking hits with RNAz score >0.5, or just those with RNAz score >0.9. The average length of an intron without predicted secondary structure is 89 bp; the average length of an intron with predicted secondary structure is 141 bp.

### Preference for predicted secondary structures to be located just inside exon boundaries

**Figure 4:**
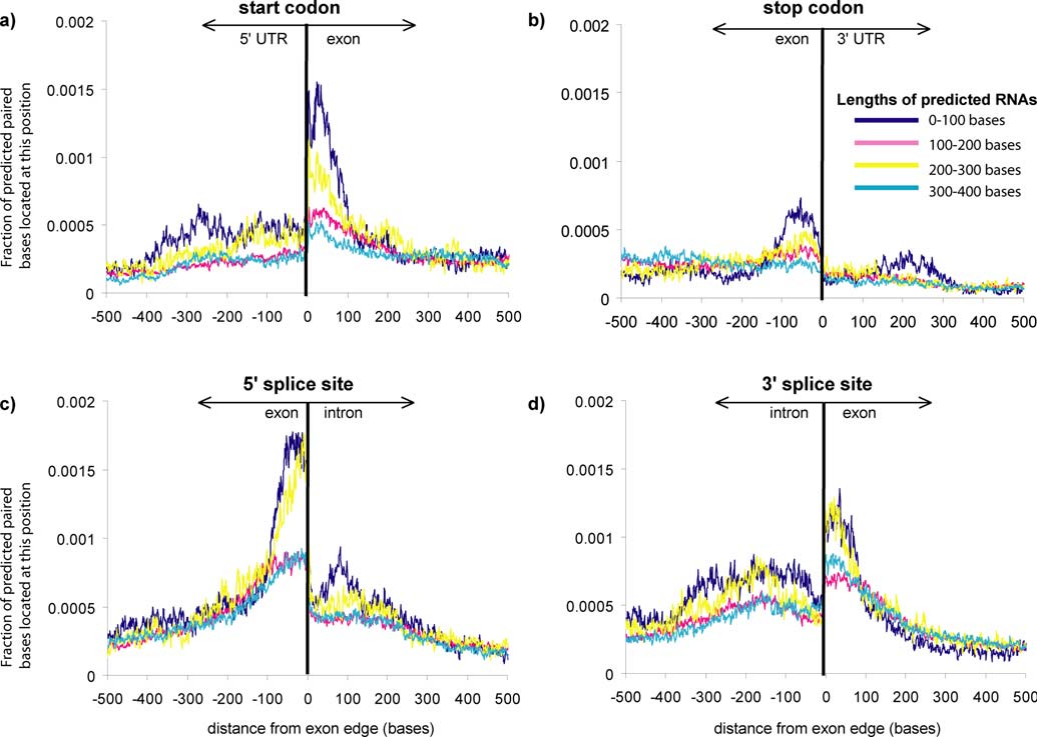
Predicted base pairs are preferentially found just inside exon boundaries. Locations of predicted base pairs were tabulated separately for four length categories of motifs (dark blue = 0–100 base long motifs, pink = 100–200 bases, yellow = 200–300 bases, light blue = 300–400 bases). These locations of predicted base pairing are plotted near the a) start codon; b) stop codon; c) 5′ splice site; and d) 3′ splice site. Predicted base-pairs involved in secondary structure are most common just inside exon boundaries, and many of these base-pairs are contained in short predicted secondary structures (0–100 bp).

**Figure 5:**
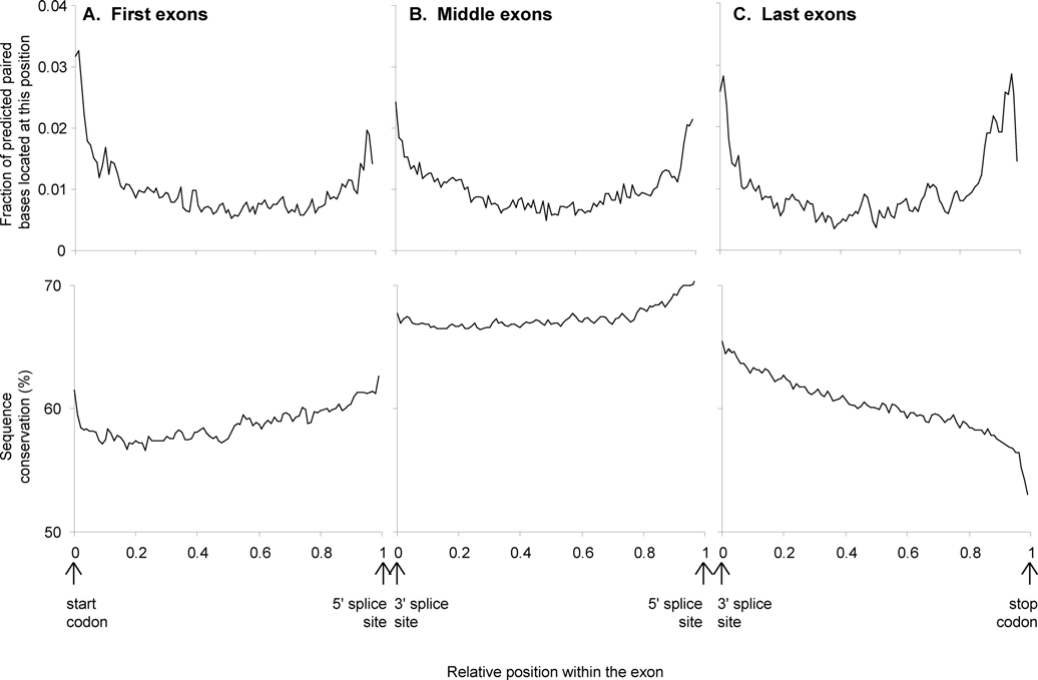
The pattern of sequence conservation near exon boundaries cannot explain the secondary structure peak just inside exon boundaries. The relative position within the exon is plotted versus the fraction of predicted base-pairs and sequence conservation for a) 5′-most exons; b) internal exons; and c) 3′-most exons. The peak in predicted secondary structure inside the exon boundary is present regardless of whether sequence conservation rises or drops near the exon boundary.

We observe an enrichment of predicted base-pairs just inside the exon boundaries (near the start codon, stop codon, 5′ splice site, or 3′ splice site; see Figure 4). This effect can not be completely explained by variations in sequence conservation near the boundary regions (see Figure 5). For 5′-most exons and middle exons, there is a rise in sequence conservation near both ends of the exon. However, last exons show a drop in sequence conservation at their 3′ end, but still exhibit an increase in predicted secondary structure at their 3′ end. (This enrichment for secondary structure just inside exon boundaries can be observed for both RNAz cutoffs of both 0.5 and 0.9.) This effect is accentuated for shorter motifs (length<100 bp), which have their predicted base pairs more concentrated towards exon boundaries than longer motifs (See Figure 4).

For 3′ and 5′ splice sites (Figure 4c and 4d), there is a sharp enrichment of predicted base pairs just inside the exon, and then another broader secondary structure peak approximately 100 bp away, on the intron side of the boundary. This second, broader region of secondary structure enrichment is due to the secondary structure peak just inside the next exon, at the other end of the intron. This broader region of secondary structure is not as sharply defined because of the variable length of the intervening intron.

**Figure 6:**
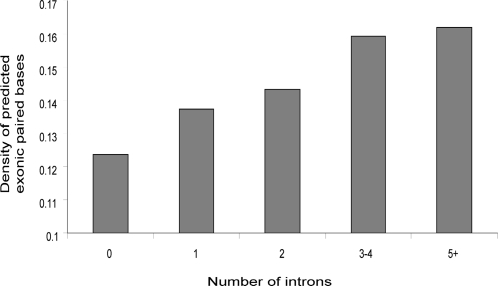
Density of predicted exonic secondary structure increases with the number of introns. The density of predicted paired bases within exons increases with the total number of introns in the gene.

We also observe an increase in the density of predicted secondary structure within exons of mRNAs containing more introns (See Figure 6). This is probably due to the fact that, as the number of introns increases, the average length of an exon decreases. Since exon edges are associated with a secondary structure peak, the density of such secondary structure peaks is increased in genes with more introns, resulting in a greater density of secondary structure in genes with more introns. The size of the secondary structure peak just inside the exons is the same for genes with one or more than one exon, so the increase is due to the increased number of peaks. Interestingly, Katz and Burge [47] looked computationally at secondary structure in bacterial coding regions and found that genes with introns had greater bias towards forming short, local secondary structures than intronless genes.

Experiments have shown that hairpins located just downstream of the start codon can compensate for suboptimal start codon context and increase translational efficiency [60]. Hairpins just downstream of the start codon have also been implicated in general cellular translational control in certain organisms [61]. Kochetov et al. have recently published a tool called AUG_hairpin designed to locate such hairpins, preferentially found at base positions 13–17 downstream of the start codon [62]. Our results show that this sort of hairpin is widespread in aspergillus, although the location downstream is broader (see Figure 4).

To further examine what sorts of motifs are found in these peaks at the edges of exons, we clustered predicted secondary structures found only in the first and last 10% of exons by structural similarity. The largest structural classes found were short hairpins and variations on short hairpins (see Figure 2e). Similar structural classes were obtained for 5′ and 3′ ends of genes, and 5′ and 3′ splice sites.

### Conclusions

We have performed a computational search for functional RNAs across a whole-genome alignment of six *Aspergillus* genomes, and clustered the resulting predictions by structural similarity. We identify a novel, ∼60 bp long hairpin motif in 86 introns. We find no evidence of microRNAs in *Aspergillus*. 3′ UTRs contain very little secondary structure compared to other regions. 5′ UTRs contain groupings of short hairpins, which are biased to lie within 50–100 bp of the start codon. We find that introns contain a great deal of secondary structure, and we show that the density of predicted intronic RNAs increases with the length of introns.

We find that predicted paired bases are most common just downstream of the start codon and 3′ splice site, and just upstream of the stop codon and 5′ splice site (just inside all types of exon boundaries). It appears that this effect is not due simply to sequence conservation within these boundary regions. The motifs found in these regions are short hairpins. We also find a surprising amount of long RNA structures within exons (primarily long, branched hairpins). The density of predicted RNA secondary structure within exons increases with the number of introns in a gene, probably because of the increased number of exonic boundary regions enriched for secondary structure near the additional splice sites.

Despite our estimates of our false positive rate (approximately 30–40% for RNAz score >0.5 and 17–21% for RNAz score >0.9), it is not clear what fraction of our predicted secondary structures are real because of difficulties in calculating false positives using shuffled controls. The real false positive rate is likely to be substantial, and further experimental work is necessary to more accurately characterize the number of functional RNAs in these fungi. It is clear that computational methods for finding and predicting functional RNAs lag behind methods for predicting protein-coding genes, and will be the subject of further development. However, RNAz was able to identify the majority of known RNAs that were contained in our alignments. And in agreement with recent results in the human genome [8], [9], it is clear that there is a large quantity of conserved RNAs of unknown function in these *Aspergillus* genomes, including several interesting specific predictions.

## Supporting Information

Figure S1 Unshuffled clusters have lower p-values than shuffled clusters. After clustering, p-values were computed for over-representation for certain genomic regions (introns, exons, etc.). These p-values were much lower for clusters made from unshuffled hits than those made from shuffled hits. The tail of the distribution displayed (low p-values) is much longer for the unshuffled hits.
(0.61 MB TIF)

Table S1 Strand Bias of RNAz hits.
(0.04 MB DOC)
