# Supplementary figures and images for: Conserved Secondary Structures in Aspergillus
Source: PLoS One. 2008 Jul 30;3(7):e2812. doi: 10.1371/journal.pone.0002812 (PMC2467506; doi:10.1371/journal.pone.0002812)

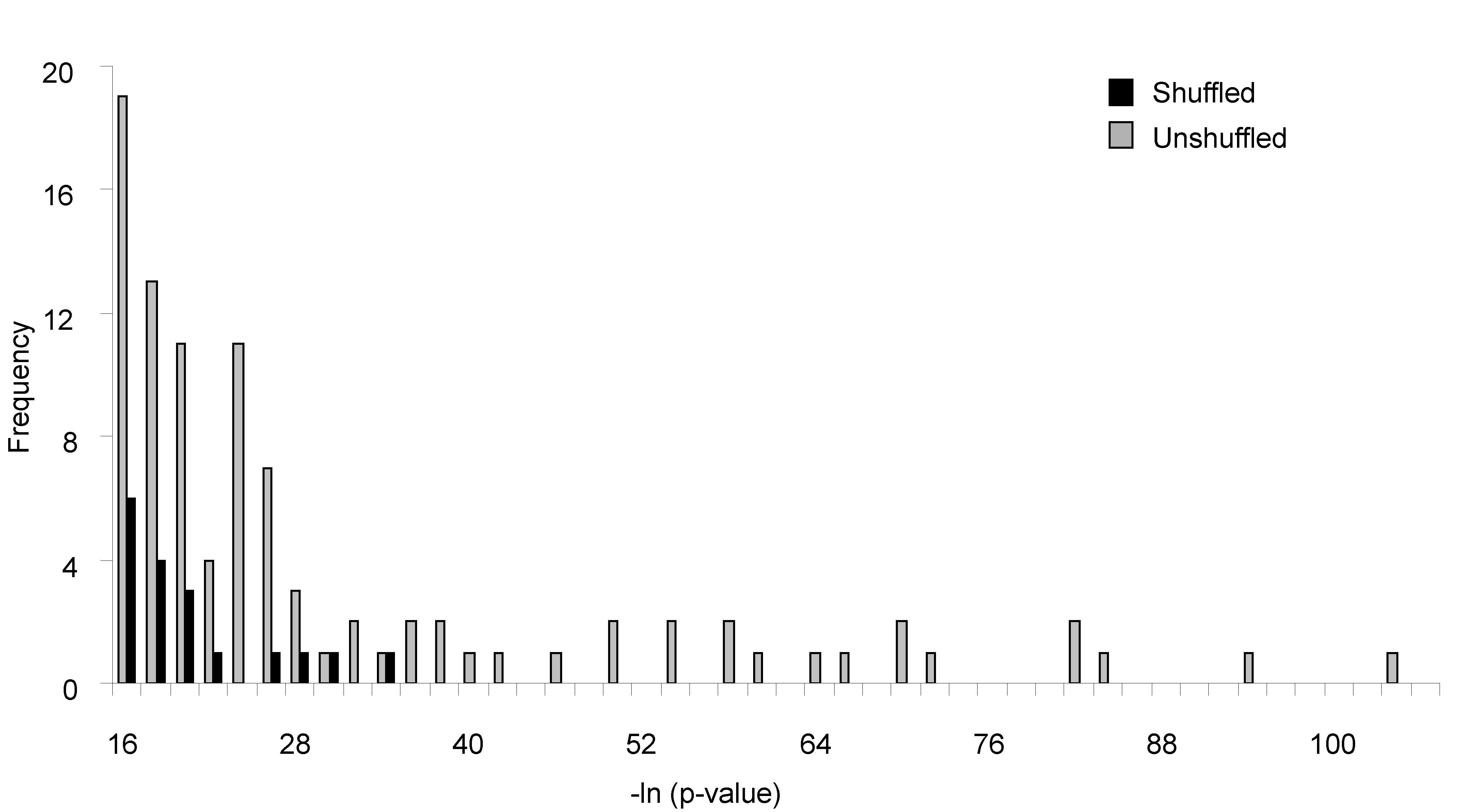

Supplement: Figure S1 — Unshuffled clusters have lower p-values than shuffled clusters. After clustering, p-values were computed for over-representation for certain genomic regions (introns, exons, etc.). These p-values were much lower for clusters made from unshuffled hits than those made from shuffled hits. The tail of the distribution displayed (low p-values) is much longer for the unshuffled hits. (0.61 MB TIF) [file pone.0002812.s001.tif]
