# Supplementary material for: Conserved Secondary Structures in Aspergillus
Source: PLoS One. 2008 Jul 30;3(7):e2812. doi: 10.1371/journal.pone.0002812 (PMC2467506; doi:10.1371/journal.pone.0002812)
Supplement: Table S1 — Strand Bias of RNAz hits. (0.04 MB DOC) [file pone.0002812.s002.doc]

**Supplementary Tables**

**Table S1. Strand bias of RNAz hits.**

|  | RNAz score >0.5 | | RNAz score >0.9 | |
| --- | --- | --- | --- | --- |
| a. 200 bp windows | Strand  bias^2^ | Strand bias  p-value  ($\chi$^2^ test)^3^ | Strand  bias^2^ | Strand bias  p-value  ($\chi$^2^ test) ^3^ |
| Intron | 1.15 | 2.0e-1 | 1.14 | 5.3e-1 |
| overlaps splice site | 1.15 | 4.3e-3 | 1.20 | 8.9e-2 |
| Noncoding | 0.89 | 1.2e-2 | 0.75 | 5.9e-3 |
| Exon | 1.03 | <1e-5 | 1.27 | 1.6e-2 |
| 5’ UTR | 1.23 | 3.0e-5 | 1.21 | 5.8e-2 |
| 3’ UTR | 1.06 | 4.3e-1 | 0.72 | 2.7e-2 |
| overlaps start | 1.66 | <1e-5 | 2.29 | <1e-5 |
| Overlaps stop | 1.11 | 2.9e-1 | 1.00 | 1 |
| Totals | 1.12 | <1e-5 | 1.15 | 1.0e-3 |
| b. 400 bp windows |  |  |  |  |
| Intron | 1.25 | 1.0e-5 | 1.75 | 2.0e-1 |
| overlaps splice site | 1.15 | 2.1e-3 | 1.49 | 5.7e-3 |
| Noncoding | 0.76 | 3.0e-4 | 0.55 | 3.0e-4 |
| Exon | 1.08 | 2.1e-1 | 0.90 | 5.5e-1 |
| 5’ UTR | 1.26 | 4.0e-5 | 1.49 | 3.0e-2 |
| 3’ UTR | 0.95 | 5.5e-1 | 0.67 | 7.7e-2 |
| overlaps start | 1.43 | <1e-5 | 2.41 | <1e-5 |
| Overlaps stop | 1.32 | <1e-5 | 0.87 | 5.2e-1 |
| Totals | 1.16 | 1e-5 | 1.22 | 1.1e-3 |
